# Growth Performance, Digestive Capacity, and Transcriptomic Analysis of the Hybrid Offspring of *Mastacembelus armatus* × *Mastacembelus favus*

**DOI:** 10.3390/ani16010011

**Published:** 2025-12-19

**Authors:** Yiman Chen, Linan Zhang, Xianshan Lin, Hao Sheng, Weikai Chen, Mingxiang Cui, Chong Han, Hu Shu

**Affiliations:** 1 School of Life Sciences, Guangzhou University, Guangzhou 510006, China; 1914130031@e.gzhu.edu.cn (Y.C.); 18258972329@163.com (L.Z.); 13926372979@163.com (X.L.); 13798942735@163.com (H.S.); 13415011547@163.com (W.C.); 2 Guangdong Lianyi Aquatic Science and Technology Development Co., Ltd., Kaiping 529300, China; m15089848917@163.com

**Keywords:** heterosis, transcriptome, non-additive expression, *Mastacembelus armatus*, *Mastacembelus favus*

## Abstract

Hybrid breeding is a crucial approach in aquaculture for improving stock performance. This study systematically evaluated heterosis in hybrid offspring from a cross between the zig-zag eel (*Mastacembelus armatus*) and the tire track eel (*Mastacembelus favus*). The hybrids exhibited significantly superior growth performance and a higher survival rate than both parental populations. Physiological analyses revealed distinct advantages in digestive enzyme activity and intestinal tissue structure. Liver transcriptome sequencing revealed that most of the differentially expressed genes (DEGs) in the hybrids displayed non-additive expression patterns and were significantly enriched in pathways associated with growth hormone synthesis, insulin secretion and lipid metabolism. The upregulation of *igf2* and the downregulation of *socs1* were identified as key molecular drivers of enhanced growth. This multi-level analysis, spanning from phenotype to molecular mechanisms, sheds light on the heterogeneous basis of these hybrids and provides theoretical insights and practical guidance for breeding work with zig-zag eels and related species.

## Simple Summary

This study successfully created hybrid offspring by crossing two different spiny eel species: *Mastacembelus armatus* and *Mastacembelus favus*. The results showed that these hybrid eels grew faster, survived better, and had more efficient digestion compared to the purebred offspring of either parent species. We found that the superior performance of the hybrids was linked to changes in the activity of many genes in their livers. These gene changes were mainly concentrated in pathways related to growth promotion, nutrient metabolism, and environmental adaptation. This research confirms that crossbreeding these two eel species is an effective strategy to improve production traits, providing a valuable basis for breeding better varieties.

## 1. Introduction

The zig-zag eel (*Mastacembelus armatus*), a high-value freshwater aquaculture species belonging to the Synbranchiformes order and Mastacembelidae family, is widely distributed across river systems south of the Yangtze River in China, as well as in India and Southeast Asia. Its delicate flesh texture sustains robust market demand [1]. Recent breakthroughs in artificial propagation technology have facilitated the rapid expansion of zig-zag eel cultivation. However, germplasm degeneration has become an increasingly prominent issue, with over-dependence on wild broodstock leading to significant variation in fry quality [2]. Therefore, developing elite zig-zag eel varieties with superior traits is crucial for the sustainable growth of this aquaculture sector.

Hybrid breeding is an effective strategy for addressing germplasm degeneration, which is defined as the interbreeding of teleosts with distinct genotypes [3]. Prolonged inbreeding in fish populations can lead to the manifestation of depressive traits, such as reduced growth rates, disease resistance and reproductive capacity [4]. Inter-strain or interspecific heterosis significantly enhances traits including growth performance, digestive efficiency, and pathogen resilience in offspring [5,6]. This approach has been shown to be highly effective in key economic fish families such as Cyprinidae, Cichlidae, Serranidae and Acipenseridae [7,8]. In the field of *M. armatus* breeding, the research group led by Shu Hu has conducted pioneering exploratory work in areas such as genetic diversity, nutritional enhancement, and disease control [9,10,11]. For example, Yang et al. [12] successfully achieved intraspecific hybridization between different geographical populations of *M. armatus* and observed heterosis in traits such as growth rate in the offspring. This provides crucial preliminary evidence for the genetic improvement of this species. However, this study also revealed that the extent of heterosis in existing intraspecific hybrid combinations is limited and that trait expression is not sufficiently stable. To achieve significant breakthroughs in breeding, the selection of parental stocks should be expanded from intra-specific geographical populations to include more distantly related species, thereby integrating a richer set of superior genes and generating more substantial heterosis. Nevertheless, this approach has not yet been explored in zig-zag eel breeding. Notably, the closely related reticulated tire track eel (*M. favus*) exhibits distinctive reticulated pigmentation and geographical adaptations [13,14], potentially offering complementary genetic resources for hybridization programs. The Tire track eel was selected as a parental species not only because of its genetic proximity to M. armatus, but also because informal pilot breeding trials conducted by local farmers at our aquaculture base prior to formal experimentation demonstrated that hybrids bred with *M. favus* as a parent exhibited good survival rates and growth vigor under practical farming conditions. While these preliminary observations were not quantified initially, they provide compelling justification for a systematic scientific investigation into its hybrid breeding potential.

The genetic mechanisms underlying heterosis in teleosts are not fully understood. Current explanatory frameworks encompass dominant complementation, overdominance and epistasis [15,16]. Growth-related traits (e.g., body weight increase and growth rate) and digestive efficiency are key indicators of heterosis, frequently correlating with metabolic pathway activation and enhanced nutrient assimilation [17]. Transcriptomic profiling has emerged as a pivotal methodology that precisely characterizes molecular-level distinctions in study subjects [18,19]. Transcriptome investigations reveal that the non-additive expression patterns of genes associated with growth, particularly those that regulate cellular proliferation and energy metabolism, are the predominant drivers in hybrid progenies [20,21]. While these findings provide valuable insights into heterosis in fish, research specific to mastacembelid fish remains understudied.

This study used zig-zag eels from the Tanjiang River basin and tire-track eels from the Lancang River basin to create four hybrid groups through complete diallel crossing. Growth performance, digestive enzyme profiles and gastrointestinal histoarchitecture were systematically compared between hybrid and purebred progenies. Liver transcriptomes were then analyzed to identify genes and metabolic pathways associated with growth heterosis that were differentially expressed. This integrated approach aimed to: (i) evaluate the breeding potential of these hybrid combinations, and (ii) elucidate the molecular basis of heterosis in mastacembelid fish.

## 2. Materials and Methods

### 2.1. Broodstock Reproduction and Management

The experimental fish were sourced from the Lianyi Aquatic Base in Jiangmen, Guangdong Province. On 2 June 2024, healthy broodstock with good physiological conditions were selected for mating. Prior to breeding, the culture ponds were disinfected, refilled with fresh water, and aerated for two days. After the broodstock acclimated, hybridization experiments were initiated. A total of 32 broodstock individuals were used, including 8 females and 8 males of the reticulated *M. favus* (LC) from the Lancang River population, and 8 females and 8 males of *M. armatus* (TJ) from the Tanjiang River population. These individuals were paired into four mating groups. Females were injected with HCG (Human Chorionic Gonadotropin) into the dorsal muscle at a total dose of 1000 IU per kg body weight for induced breeding. The hormone was administered in two injections: the first one was 20% of the total dose, and the second one, comprising the remaining 80%, was given 24 h later. The fish were then kept in a hatching tank at 28 °C. After 45 h, manual egg stripping was performed. The eggs from each population were divided into two equal portions. Similarly, semen was manually collected from males and also divided into two equal portions. Artificial fertilization was then conducted according to the following four crossing combinations:

LC♂ × TJ♀ (*M. favus*♂ × *M. armatus*♀, abbreviated as LT);
TJ♂ × LC♀ (*M. armatus*♂ × *M. favus*♀, abbreviated as TL);
TJ♂ × TJ♀ (*M. armatus* pure crossing, abbreviated as TJ);
LC♂ × LC♀ (*M. favus* pure crossing, abbreviated as LC).

The offspring were hatched in four plastic tanks measuring 1.2 m × 0.9 m × 0.7 m. To minimize early environmental bias, the fry from each genetic group were initially reared in three replicate plastic tanks until 30 days post-hatching. At 30 days post-hatching, due to constraints in space and resources at the aquaculture facility, the fry from the three replicate tanks within each genetic group were pooled and transferred to a single net cage (2.0 m × 2.0 m × 1.5 m) for the remaining rearing period. Thus, each group (TJ, LC, LT, TL) was ultimately reared in one net cage during the experimental grow-out phase. All four net cages were placed in the same concrete pond to standardize overarching environmental conditions such as water source and major water quality parameters. The water temperature was maintained at 28.5 ± 1.0 °C, pH at 7.18 ± 0.45, and dissolved oxygen at 6 ± 0.5 mg/L. The four groups of offspring were subjected to identical feeding and management regimens, including feed formulation and feeding strategies (Supplementary Tables S1 and S2).

### 2.2. Growth Performance Comparison

Growth performance of *M. armatus* offspring at 30, 60, and 90 days of age were generated from measurements conducted on 7 July, 6 August, and 5 September. The initial stocking density for each genetic group at the time of transfer to net cages (30 days post-hatching) was 400 individuals. At each measurement time point, a random sample of 30 fish was drawn from each net cage for measurement of body length (mm) and body weight (g). Specific growth rates for length (*SGR_L_*) and weight (*SGR_W_*), as well as the survival rate (*SR*), were calculated using the following formulas:

*SGR_L_* = (ln*L*_2_ − ln*L*_1_)/*T* × 100%

*SGR_W_* = (ln*W*_2_ − ln*W*_1_)/*T* × 100%

*SR* = Final fish number/Initial fish number × 100%

where *L*_1_ and *W*_1_ represent initial body length (mm) and weight (g), *L*_2_ and *W*_2_ the final measurements, and *T* the culture period in days. Hybridization effects on growth performance were analyzed using one-way ANOVA with significance set at *p* < 0.05.

### 2.3. Sample Collection

At 90 days post-hatching, offspring of *M. armatus* were fasted for 24 h. A total of 12 fish per group were randomly sampled for subsequent biochemical and histological analyses. Specifically, these 12 fish were divided into three subsets: 4 for digestive enzyme assays (Section 2.4), 4 for intestinal histology (Section 2.5), and 4 for transcriptome sequencing (Section 2.6). All fish were captured with fishing nets, anesthetized in 0.1 mL/L eugenol solution, euthanized via immediate decapitation, and dissected. Tissue samples (liver, intestine, stomach) were collected, snap-frozen in liquid nitrogen, and stored at −80 °C.

### 2.4. Digestive Enzyme Activity Assays

Intestinal, gastric, and hepatic tissues from 4 individual fish per group (*n* = 4 biological replicates) were analyzed separately. For each fish, 0.1 g each of intestinal, gastric, and hepatic tissues was homogenized in 1.0 mL of ice-cold 0.8% saline solution for 3 min on ice. Homogenates were centrifuged at 2500× *g* for 10 min, and 500 µL of supernatant was collected for enzyme activity measurement. Analyzed enzymes included: pepsin, trypsin, lipase, and amylase.

All assays were performed using specialized kits (Nanjing Jiancheng Bioengineering Institute) strictly following the manufacturer’s instructions. Enzyme activity values reported in this study represent specific activity, expressed as units per milligram of protein (U/mg protein). The protein concentration of the supernatant was determined to facilitate this calculation [Bicinchoninic Acid Assay].

The assays were performed strictly following the manufacturer’s instructions. The general principle underlying the kits involves measuring the rate of substrate conversion catalyzed by the enzyme under optimized conditions.

Pepsin and Trypsin activity were determined by measuring the hydrolysis of specific protein substrates, with the resulting product quantified colorimetrically. Amylase activity was assessed based on its ability to hydrolyze starch, and the reaction rate was determined by measuring the resulting change in absorbance. Lipase activity was measured by monitoring the hydrolysis of lipid substrates, with the generation of fatty acids or other products being quantified.

According to the manufacturer’s specifications, one unit (U) of enzyme activity is defined as the amount of enzyme required to catalyze the conversion of 1 μmol of substrate per minute under the defined assay conditions. Absorbance was measured using a microplate reader, and enzyme activities were calculated based on the standard curves provided with the kits.

### 2.5. Intestinal Histology Preparation and Observation

Fresh intestinal tissues were collected from four fish per group. The mid-intestinal segments were dissected and fixed in 4% paraformaldehyde solutions for 24 h. Subsequently, the tissues were dehydrated through a graded ethanol series, cleared in xylene, and embedded in paraffin. Transverse sections of 5 µm thickness were prepared and stained with Hematoxylin and Eosin (H&E). The stained sections were observed and imaged under an optical microscope. Intestinal structural differences were analyzed using Image J software (version 1.53t, National Institutes of Health, Bethesda, MD, USA).

All histological assessments were performed independently by an investigator blinded to the experimental group assignments. Measurements included villus height, villus width, crypt depth, and muscularis thickness. For each biological replicate within a treatment group (i.e., 4 fish per group), at least 15 intact and well-oriented villi were randomly selected for measurement (n ≥ 15). The measurement of the intestinal mucosal perimeter was conducted on three representative cross-sections (*n* = 3) selected from each group.

Measurements included villus height, villus width, crypt depth, and muscularis thickness. Mucosal length was measured by tracing the mucosal contour along intestinal cross-sections. Villus density was calculated as follows: Villus density (villi/mm) = Number of villi/Corresponding mucosal length.

### 2.6. Transcriptome Sequencing

To investigate the molecular mechanisms underlying heterosis in *M. armatus*, transcriptome sequencing was performed on four offspring groups (TJ, LC, TL, LT). To ensure adequate statistical power for differential expression analysis, four biological replicates per group were included Total RNA was isolated from 200 mg of liver tissue collected from each biological replicate.

Liver total RNA was extracted using TRIzol reagent (Vazyme Biotech Co., Ltd., Nanjing, China), followed by digestion with DNase I (TaKaRa Bio Inc., Shiga, Japan) to eliminate genomic DNA contamination. RNA quality was rigorously assessed: integrity was confirmed by 1.0% agarose gel electrophoresis, the RNA Integrity Number (RIN) was determined using an Agilent 2100 Bioanalyzer (Agilent Technologies, Santa Clara, CA, USA), and purity was verified with a NanoDrop 2000 spectrophotometer (Thermo Fisher Scientific, Waltham, MA, USA) (OD_260/280_ = 1.8–2.2, OD_260/230_ > 2.0). Only high-quality RNA samples (RIN > 7) were used for subsequent library construction. Equal amounts of RNA from each biological replicate within a group were pooled, and the pooled RNA was then used to construct cDNA libraries.

Paired-end sequencing (2 × 150 bp) was performed using the NovaSeq 6000 platform (Illumina Inc., San Diego, CA, USA). Raw sequencing data underwent quality control using Perl scripts (version 5.32.1, The Perl Foundation, Walnut, CA, USA), which assessed Q20, Q30, and GC content. High-quality clean reads were retained for downstream analysis. The clean reads were aligned to the *M. armatus* reference genome (downloaded from NCBI) using the STAR software (version 2.7.10a, Cold Spring Harbor Laboratory, NY, USA). Read counts per gene were quantified using HTseq (version 0.13.5, European Molecular Biology Laboratory, Heidelberg, Germany), and gene expression levels were normalized as FPKM (Fragments Per Kilobase of transcript per Million mapped reads). Differential expression analysis was conducted using DESeq2 (R package, version 1.38.3, Bioconductor) to perform pairwise comparisons between groups, with significantly differentially expressed genes (DEGs) defined as those exhibiting |log2 fold change| > 1 and an FDR (False Discovery Rate) < 0.05. To enhance the reliability of the DEG identification, an independent validation was performed using edge R (R package, version 3.40.2, Bioconductor), requiring |log2 fold change| > 1 and an adjusted *p*-value < 0.05 for significance. This dual approach employing both DESeq2 and edge R aligns with best practices for improving the robustness of DEG detection [22].

### 2.7. Additive and Dominance Effect Analysis

Gene expression patterns were analyzed according to the method described by Ryan et al., which categorized the differential expression of hybrid offspring (groups LT and TL) relative to their parents (groups TJ and LC) into twelve distinct patterns [23]. First, the average expression value of the two parents was calculated to obtain the mid-parent expression value (MPV). Expression patterns in the hybrid offspring approximating the MPV were classified as additive expression. Patterns significantly deviating from the MPV (*p* < 0.05) were categorized as non-additive expression [24]. Gene Ontology (GO) and Kyoto Encyclopedia of Genes and Genomes (KEGG) enrichment analyses were performed on genes exhibiting non-additive expression using the cluster Profiler R package (version 4.6.2, Bioconductor) to investigate their biological functions.

### 2.8. Validation of Transcriptome Results by qRT-PCR (Quantitative Real-Time PCR)

Total RNA was isolated from liver tissue using TRIzol reagent. Residual genomic DNA contamination was then removed using the gDNA wiper kit (Vazyme Biotech Co., Ltd., Nanjing, China). The integrity and concentration of the RNA were assessed via 1.0% agarose gel electrophoresis and verified using a microplate reader (BioTek Instruments, Winooski, VT, USA). Four biological replicates were prepared per group (LC, TJ, LT and TL). Reverse transcription was performed using HiScript II RT SuperMix (Vazyme Biotech Co., Ltd., Nanjing, China)to generate cDNA. The reaction mixture contained 8 µL of HiScript II RT Super Mix, 1 µg of total RNA template and nuclease-free ddH_2_O to bring the final volume to 20 µL. The thermal cycling protocol consisted of an incubation step at 37 °C for 15 min, followed by enzyme inactivation at 85 °C for 5 s.

qRT-PCR was performed using an ABI 7900 HT system (Thermo Fisher Scientific, Waltham, MA, USA) and β-actin was used as the endogenous control gene. Ten randomly selected DEGs underwent validation [25]. Primers were designed using Primer Premier 5.0 (Premier Biosoft, Palo Alto, CA, USA) and synthesized commercially by Sangon Biotech (Shanghai, China).

Reactions utilized a 20 μL mixture containing: 10 μL ChamQ Universal SYBR qPCR Master Mix (Vazyme, China), 0.4 μL each of forward and reverse primers (10 μM), 1 μL cDNA template, and 8.2 μL nuclease-free ddH_2_O. Thermal cycling comprised initial denaturation at 95 °C for 30 s, followed by 40 cycles of 95 °C for 10 s and 60 °C for 30 s. Melting curve analysis was subsequently conducted through sequential incubations at 95 °C for 10 s, 60 °C for 60 s, and 95 °C for 15 s. Relative gene expression quantification employed the 2^−ΔΔCT^ method [26].

### 2.9. Statistical Analysis

Data were analyzed using SPSS 24.0 software (SPSS Inc., Chicago, IL, USA). Data were analyzed using SPSS 24.0 software (SPSS Inc., USA). After a significant result from one-way analysis of variance (one-way ANOVA), Tukey’s Honestly Significant Difference (HSD) post hoc test was applied for all pairwise comparisons between groups to control the family-wise error rate. Statistical significance was defined as *p* < 0.05. Figures for qRT-PCR results were generated using GraphPad Prism software (version 9.0.0, GraphPad Software, San Diego, CA, USA).

## 3. Results

### 3.1. Growth Performance Variations in Zig-Zag Eel Groups

Phenotypic analysis of skin patterning revealed distinct differences between the offspring of the LC and TJ inbred groups, characterized by clear reticulated patterns and blurred, python-like patterns, respectively. In contrast, the hybrid offspring of both LT and TL crosses exhibited a consistent phenotype: they possessed the reticulated patterning on the dorsal side, similar to the LC parent, while the ventral side, which is patternless in the TJ parent, displayed vertical striations. This combined pattern suggests a transitional state intermediate between the two parental types (Supplementary Figure S1).

**Figure 1:**
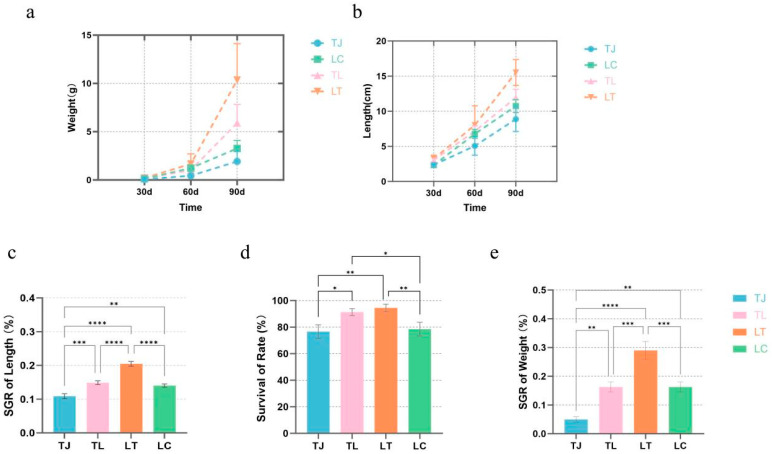
(**a**) Body length growth curves of four zig-zag eel offspring groups over three months; (**b**) Body weight growth curves of four zig-zag eel offspring groups over three months; (**c**) Specific growth rate (*SGR*) for body length in four zig-zag eel offspring groups; (**d**) Survival rate of four zig-zag eel offspring groups; (**e**) Specific growth rate (*SGR*) for body weight in four zig-zag eel offspring groups. Data are presented as mean ± SD. Asterisks indicate statistically significant differences between groups (* *p* < 0.05, ** *p* < 0.01, *** *p* < 0.001, **** *p* < 0.0001).

During the three-month culture period, all four zig-zag eel groups-TJ purebred, LC purebred, LT hybrid progeny, and TL hybrid progeny-exhibited continuous increases in growth phenotypes (body length and body weight) (Figure 1a,b). Statistical analyses demonstrated significantly superior growth performance in both hybrid progeny groups (LT and TL) compared to the parental purebred groups (TJ and LC). All data from the above groups satisfied the normal distribution (Supplementary Table S4).

To quantitatively compare growth differentials, specific growth rates based on length (*SGR_L_*) and weight (*SGR_W_*) were calculated. A one-way ANOVA revealed significant intergroup differences for both *SGR_L_* (F(3, 56) = 42.05, *p* < 0.001) and *SGR_W_* (F(3, 56) = 22.74, *p* < 0.001) (Figure 1c,e). The LT hybrid progeny displayed the highest *SGR* values, while the TJ purebred group showed significantly lower *SGR* than the other three groups. Furthermore, survival rates of LT and TL hybrid progenies significantly exceeded those of TJ and LC purebred groups (F (3, 8) = 14.14, *p* = 0.0015) (Figure 1d) (Supplementary Table S3).

### 3.2. Quantification of Digestive Enzyme Activities

**Figure 2:**
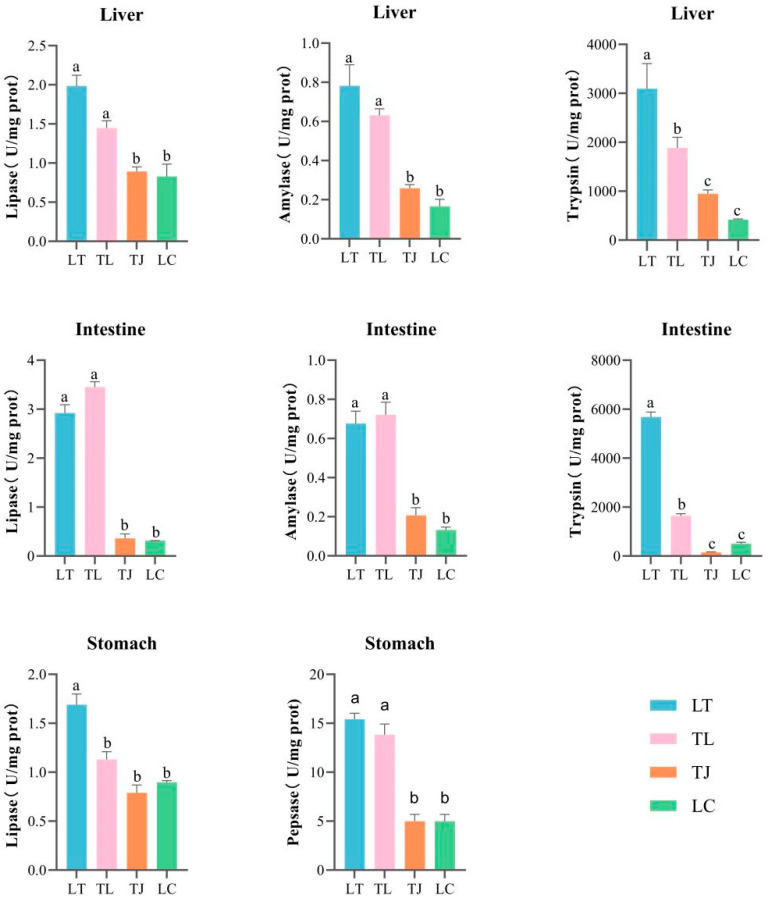
Comparison of digestive enzyme activities across intestinal, hepatic, and gastric tissues in four zig-zag eel offspring groups. Different superscript letters for data in the same row indicate significant differences (*p* < 0.05).

Statistical analyses of digestive enzyme activities (amylase, lipase, trypsin, pepsin) in the intestine, liver, and stomach of zig-zag eel were conducted using one-way ANOVA followed by Tukey’s HSD post hoc test (Figure 2). All data from the above groups satisfied the normal distribution (Supplementary Table S6). A consistent hierarchical gradient was observed across most enzymatic parameters, where the LT hybrid progeny exhibited the highest activity levels, followed sequentially by TL hybrid progeny > TJ purebred > LC purebred (*p* < 0.05) (Supplementary Table S5). The overall digestive enzyme activities in both hybrid groups significantly surpassed those in purebred groups. Notably, two exceptions to this pattern emerged: intestinal amylase and lipase activities were significantly higher in the TL hybrid progeny than in the LT group (*p* < 0.05), while gastric lipase activity showed no significant difference between TL hybrid progeny and either purebred group (*p* > 0.05).

### 3.3. Histomorphometric Analysis of Intestinal Tissues

**Figure 3:**
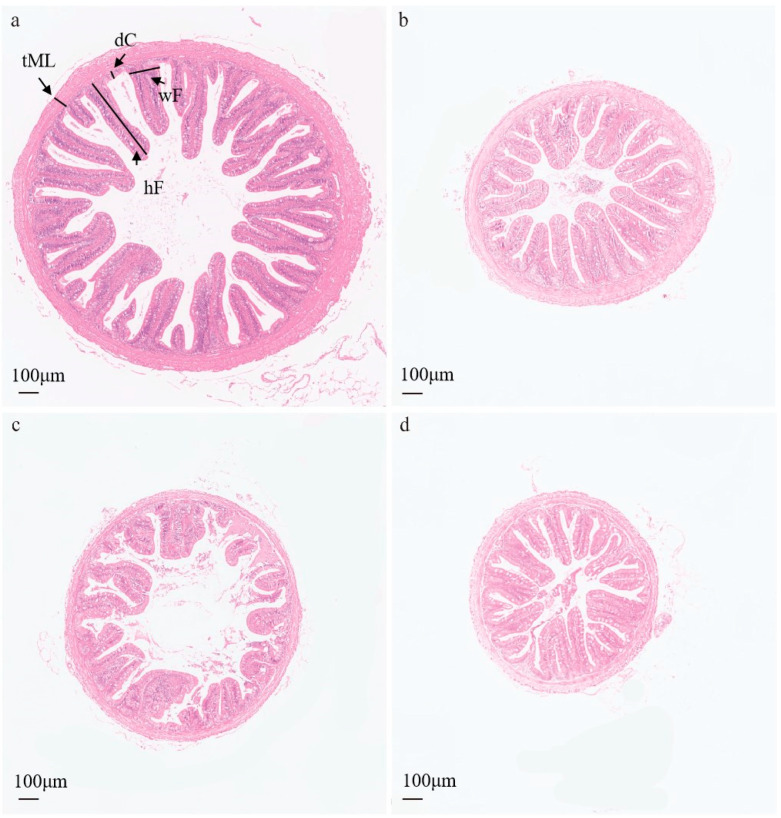
(**a**) Representative intestinal photomicrograph of LT group zig-zag eel; (**b**)Representative intestinal photomicrograph of TL group zig-zag eel; (**c**) Representative intestinal photomicrograph of TJ group zig-zag eel; (**d**) Representative intestinal photomicrograph of LC group zig-zag eel. Abbreviations: dC, crypt depth; wF, fold width; hF, fold height; tML, muscle thickness.

Histological assessment of midgut sections demonstrated well-organized, densely arranged intestinal folds in LT hybrid progeny, TL hybrid progeny, and LC purebred, while TJ purebred exhibited comparatively sparse and shortened folds (Figure 3). Subsequent morphometric quantification revealed significantly greater fold length in both hybrid groups relative to purebred groups (*p* < 0.05). However, fold width showed no significant intergroup variation. For fold density and muscular thickness, TL hybrid progeny registered maximal values that significantly surpassed other groups, with LT hybrid progeny and LC purebred also showing significantly higher values than TJ purebred. Hybrid groups exhibited significantly deeper crypts than purebred groups (Table 1). The fold length-to-crypt depth ratio varied significantly among groups, peaking in LT hybrid progeny followed sequentially by TL hybrid progeny ≈ LC purebred > TJ purebred (*p* < 0.05).

### 3.4. Differential Transcriptome Profiling

**Figure 4:**
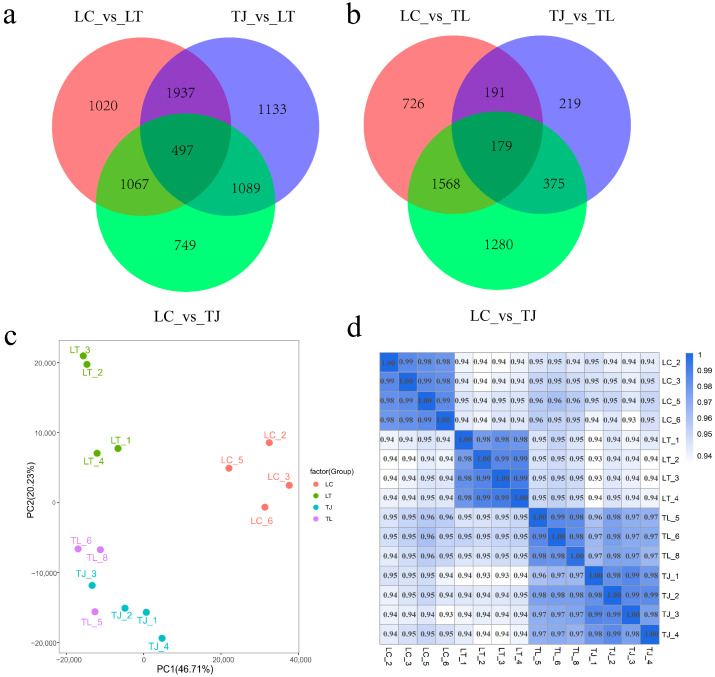
(**a**) Venn diagram of differentially expressed genes (DEGs) in hepatic tissues among LT, TJ, and LC groups of zig-zag eel; (**b**) Venn diagram of DEGs in hepatic tissues among TL, TJ, and LC groups; (**c**) Principal component analysis (PCA) of RNA-seq data from four zig-zag eel groups; (**d**) Hierarchical clustering heatmap of hepatic gene expression profiles across individual fish in four groups.

The differential expression results of qRT-PCR including 10 DEGs (*igf2*, *dio2*, *slc5a5*, *gskcb*, *raf1*, *elovl7*, *pdx1*, *socs1*, *igfbp2*, *lepr*) were consistent with RNA-seq. Liver transcriptome sequencing data quality metrics are presented in Table 2. Post-quality control, hepatic samples showed mean GC content of 49.53% with average Q20 and Q30 scores of 98.55% and 96.07%, respectively. Alignment analysis demonstrated 67.88% of sequences successfully mapped to the zig-zag eel genome across all liver groups. Differential gene expression analysis (Figure 4a,b) identified 4521 DEGs in the LT vs. LC comparison, 4656 DEGs for LT vs. TJ, and 3402 DEGs for LC vs. TJ; intersected analysis revealed 497 genes common to all three comparisons. In analogous TL hybrid analysis, TL vs. LC showed 2372 DEGs, TL vs. TJ yielded 990 DEGs, and the LC vs. TJ comparison maintained 3402 DEGs, with triple overlap comprising 179 genes.

TPM-based expression matrices from 15 hepatic samples (Figure 4c,d) confirmed pronounced segregation of the four genotypic groups, indicating distinct transcriptomic profiles with high intergroup discrimination and experimental reproducibility.

### 3.5. Regulatory Significance of Non-Additive Expression in Hybrid Vigor

**Figure 5:**
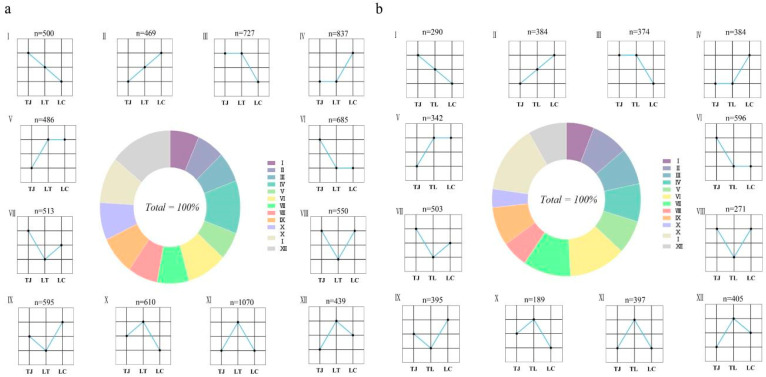
(**a**) Expression pattern profiling of differentially expressed genes (DEGs) in hepatic tissues of TJ, LC, and LT groups; (**b**) Expression pattern profiling of DEGs in hepatic tissues of TJ, LC, and TL groups. (Roman numerals I–XII denote distinct gene expression patterns).

Hepatic transcriptomes from 15 zig-zag eels were used to classify genetic expression patterns of DEGs in hybrid group LT relative to parental groups LC and TJ. Gene pairs with expression differences below |log_2_FC| < 1.25 between hybrid and either parent were defined as conserved expression. Quantitative comparison of expression divergence between LT hybrids and parental groups categorized 7475 DEGs into 12 distinct patterns. Additive expression patterns (classes I and II) accounted for 12.9% (969/7475), whereas the remaining 87.1% (6506/7475) exhibited non-additive expression (Figure 5a). Analogous analysis of TL hybrids identified 4533 DEGs, of which additive patterns represented 14.9% (674/4533) and non-additive patterns constituted 85.1% (3859/4533) (Figure 5b).

### 3.6. Functional Enrichment of Non-Additive Genes

**Figure 6:**
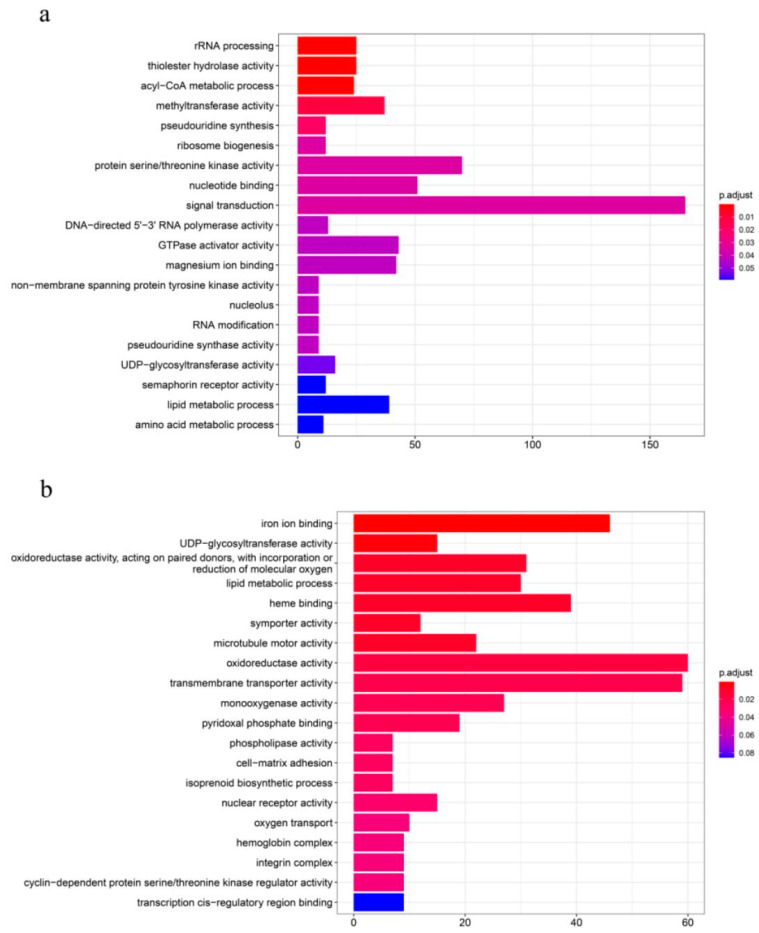
(**a**) Displaying the top 20 significantly enriched pathways for shared non-additively expressed genes in GO analyses of LT, TJ and LC comparisons; (**b**) Displaying the top 20 significantly enriched pathways for shared non-additively expressed genes in GO analyses of TL, TJ and LC comparisons.

To further investigate the mechanistic role of non-additive expressed genes in heterosis, GO enrichment analysis was conducted on 6506 non-additive genes in LT hybrids. Results demonstrated significant enrichment in molecular function (MF) categories including transmembrane transporter activity, guanyl-nucleotide exchange factor activity, protein serine/threonine kinase activity, phosphatase activity, iron ion binding, nucleotide binding, protein tyrosine kinase activity, and GTPase activator activity. Biological processes (BP) were primarily enriched in signal transduction, lipid metabolic process, protein folding, RNA processing, rRNA processing, DNA-templated transcription, acyl-CoA metabolic process, and tRNA aminoacylation, while cellular components (CC) showed enrichment in transcription regulator complex and nucleolus (Figure 6a).

Analysis of 3859 non-additive genes in TL hybrids revealed MF enrichment in iron ion binding, UDP-glycosyltransferase activity, oxidoreductase activity (acting on paired donors with incorporation/reduction of molecular oxygen), heme binding, symporter activity, and microtubule motor activity. BP categories exhibited significant enrichment in cell–matrix adhesion and oxygen transport, with CC predominantly localized to hemoglobin complex and integrin complex (Figure 6b).

### 3.7. Pathway Clustering of Non-Additive Genes

**Figure 7:**
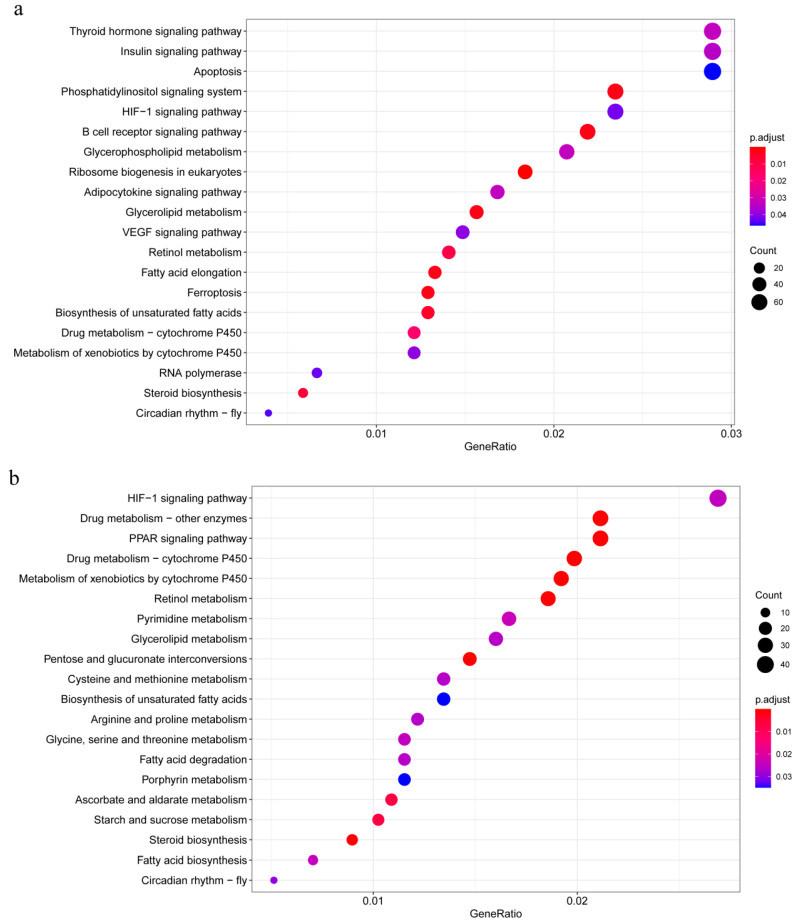
(**a**) Top 20 significantly enriched pathways for shared non-additively expressed genes in KEGG pathway analysis of LT, TJ and LC comparisons; (**b**) Top 20 significantly enriched pathways for shared non-additively expressed genes in KEGG analysis of TL, TJ and LC comparisons.

The KEGG enrichment analysis of non-additively expressed genes in the LT hybrid group (top 20 pathways) revealed predominant enrichment in three major functional categories: growth and development regulation, protein synthesis, and metabolic stress response (Figure 7a). Pathways associated with growth and development included thyroid hormone signaling, insulin signaling, as well as metabolism of growth-related substances: retinol metabolism, glycerophospholipid metabolism, and unsaturated fatty acid biosynthesis. Protein synthesis-related pathways encompassed the eukaryotic ribosome biogenesis and the RNA polymerase pathway. Metabolic stress pathways involved lipid metabolism (e.g., “Fatty acid elongation”, “Steroid biosynthesis”, “Glycerolipid metabolism”, and “Adipocytokine signaling pathway”), cytochrome P450-mediated detoxification pathways (“Drug metabolism—cytochrome P450” and “Metabolism of xenobiotics by cytochrome P450”), along with immune-related pathways (“Apoptosis”, “HIF-1 signaling pathway”, and “Ferroptosis”).

The top enriched pathways in the TL hybrid group were similarly centered on substance metabolism, including the PPAR signaling pathway, glycerolipid metabolism, unsaturated fatty acid biosynthesis, starch and sucrose metabolism, and amino acid metabolism pathways such as arginine/proline metabolism and glycine/serine/threonine metabolism. The identical cytochrome P450 detoxification pathways were also enriched, along with immune-related pathways like”Porphyrin metabolism” and “Ascorbate and aldarate metabolism” (Figure 7b).

A key finding was that compared with parental inbred groups, both LT and TL hybrid groups exhibited common differential enrichment in five pathways: HIF-1 signaling, cytochrome P450-mediated drug metabolism, xenobiotic metabolism, fatty acid degradation, and glycerolipid metabolism.

### 3.8. Quantitative Real-Time PCR Validation of Hepatic DEGs

**Figure 8:**
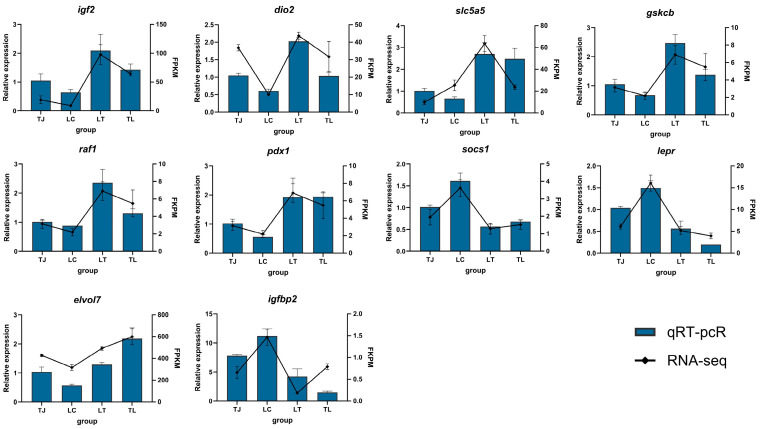
Results of liver transcriptome qRT-PCR verification. The data are presented as mean ± SEM, with the TJ inbred group serving as the control group.

To comprehensively identify potential heterosis-related candidate genes, we performed a systematic screen for non-additively expressed genes in both hybrid groups (LT and TL) relative to the two parental inbred groups. Ultimately, from differentially expressed genes (DEGs) obtained by comparing each hybrid group (LT or TL) with both inbred groups, we identified 10 non-additively expressed genes functionally important for growth and development (*igf2*, *dio2*, *slc5a5*, *gskcb*, *raf1*, *elovl7*, *pdx1*, *socs1*, *igfbp2*, *lepr*) (Figure 8).

As shown in Figure 8 (with inbred group TJ as the reference), the expression levels of *igf2*, *dio2*, *slc5a5*, *gskcb*, *raf1*, *elovl7*, and *pdx1* were consistently higher in both hybrid groups (LT and TL) than in the two inbred groups (TJ and LC), while their expression in the LC inbred group was further reduced compared to the TJ group. Conversely, *socs1*, *igfbp2*, and *lepr* exhibited lower expression levels in both hybrid groups than in the inbred groups, with higher expression in the LC inbred group relative to the TJ group.

Although quantitative discrepancies were observed between transcriptomic data and qRT-PCR validation for certain genes, their overall expression trends aligned, further validating the reliability of the transcriptome analysis.

## 4. Discussion

### 4.1. Hybrids Exhibit Superior Growth Performance

Extensive research has confirmed that heterosis, often manifested as superior growth and yield, is a common outcome of hybridization, primarily resulting from genetic complementarity between parental genomes [27,28,29]. Based on this principle, our interspecific cross between the Tanjiang zig-zag eel and the Lancang tire-track eel successfully generated hybrid offspring (LT and TL groups) that demonstrated significantly enhanced specific growth rate, weight gain, and survival compared to their purebred parental groups (TJ and LC). This indicates that heterosis in this system not only confers growth advantages but also improves robustness. The observed maternal effect—where the LT cross outperformed the reciprocal TL cross—highlights the significant influence of cross direction on trait expression, a phenomenon consistent with findings in other hybrid fish [12,30]. This directional superiority suggests the involvement of non-Mendelian mechanisms, such as mitochondrial inheritance or genomic imprinting, warranting further investigation.

### 4.2. Enhanced Digestive Physiology Underpins Hybrid Growth

The superior growth of the hybrids was strongly correlated with a systemic enhancement of their digestive capacity. Key digestive enzymes (pepsin, trypsin, amylase, lipase) exhibited significantly higher activity in the intestines of hybrids compared to purebreds, indicating more efficient nutrient hydrolysis and assimilation potential [31,32].

This physiological advantage is a common hallmark of heterosis in fish, as documented in hybrid yellow catfish and marble goby [33,34]. Importantly, this biochemical superiority was reflected in intestinal morphology. The hybrids, particularly the LT group, possessed longer and denser intestinal folds, a thicker muscular layer, and a higher villus height-to-crypt depth (V/C) ratio. These structural adaptations collectively expand the intestinal absorptive surface area and enhance intestinal motility, thereby maximizing nutrient uptake efficiency [35,36]. Similar positive correlations between intestinal morphology and growth rate have been reported in species such as *Micropterus salmoides* and hybrid crucian carp, where transcriptomic evidence links these traits to the upregulation of genes involved in cell proliferation and adhesion [37,38]. Therefore, the coordinated enhancement of both biochemical functions and structural features of the digestive system contributes to heterosis.

### 4.3. Non-Additively Expressed Genes Play Crucial Roles in Hybrid Superiority

The liver serves as a central organ for metabolism and growth regulation [39]. Transcriptomic analysis of the liver revealed that non-additive gene expression dominates, accounting for over 80% of differentially expressed genes (DEGs) in hybrids. This prevalence indicates that non-additive effects (e.g., overdominance, epistasis) are primary drivers of interspecific hybrid heterogeneity, consistent with findings in tilapia, abalone, and crayfish [40,41,42,43,44,45]. Functional analysis of enriched pathways, combined with alterations in downstream metabolic and endocrine regulatory networks, enabled us to construct a preliminary integrated model explaining the mechanisms underlying hybrid vigor formation.

First, the upregulation of lipid metabolism genes (*gskcb*, *elovl7*) may provide the material and energy foundation for rapid growth by enhancing energy storage and membrane biosynthesis capacity [46,47]. Simultaneously, the downregulation of the satiety signal gene *lepr* may prolong feeding behavior, increase nutrient intake, and indirectly promote growth [48]. Building upon this, these metabolic-level changes are further synergistically driven by endocrine system reprogramming.

On one hand, expression of growth-promoting genes increases: insulin-like growth factor 2 *igf2* shows significantly elevated expression in the hybrid group. This gene plays a crucial role in fish muscle growth and body weight regulation, directly promoting protein synthesis and cell proliferation by activating the IGF1R/Akt/MAPK signaling axis [49]. Simultaneously, the type II deiodinase gene (*dio2*) was upregulated. The enzyme it encodes catalyzes the conversion of the thyroid hormone T4 to its more active form T3, contributing to increased basal metabolic rate and growth development velocity [50]. Conversely, growth-inhibitory factors were suppressed: insulin-like growth factor-binding protein 2 (*igfbp2*) and cytokine-stimulated inhibitory factor 1 (*socs1*) exhibited reduced expression in the hybrid group. These proteins inhibit growth signaling under normal conditions by antagonizing the IGF pathway and negatively regulating the GH/IGF-I axis, respectively [51,52]. Consequently, the upregulation of *igf2* and *dio2*, combined with the downregulation of *igfbp2* and *socs1*, collectively establishes an endocrine environment conducive to anabolic processes, thereby systematically enhancing the growth capacity of hybrid individuals.

Notably, the enrichment of cytochrome P450 and HIF-1 signaling pathways provides a potential molecular mechanism for the improved survival rate of hybrids. The non-additive expression patterns of these pathways may not only optimize the endocrine environment related to growth but also directly enhance the hybrids’ ability to cope with common environmental stresses. Specifically, the cytochrome P450 enzyme system not only participates in steroid hormone metabolism to finely regulate growth [53], but also serves as a critical detoxification barrier for organisms responding to exogenous toxic substances (such as algal toxins, drug residues, or organic pollutants potentially present in aquaculture water) and oxidative stress [54,55]. The HIF-1 signaling pathway serves as the core regulatory network through which cells perceive and respond to hypoxia—a stress factor frequently encountered in aquaculture due to high stocking densities, elevated water temperatures, or deteriorating water quality [56,57].

The reshaping of gene expression in these pathways among hybrids in this study may confer enhanced detoxification capacity and hypoxic tolerance, providing a potential molecular explanation for the significantly higher survival rates observed in both hybrid groups compared to the purebred group. This advantage in stress response pathways constitutes a crucial aspect of the “robustness” within hybrid vigor, enabling sustained survival and growth even under suboptimal or fluctuating environmental conditions. From an evolutionary perspective, the widespread non-additive gene expression observed in hybrid progeny suggests that the parental species (*M. armatus* and *M. favus*) have accumulated significant cis- or trans-regulatory differences during their long periods of independent evolution. When parental genomes combine in hybrids, these divergent regulatory elements and transcription factors may form novel interactions, causing the expression levels of numerous genes to deviate from parental averages. This results in non-additive effects, such as compensatory interactions between cis- and trans-acting elements [3].

Differences in growth and digestive traits observed in backcross (LT × TL) progeny further suggest the presence of parent-of-origin effects. This may be driven by genomic imprinting (involving parent-specific gene expression) or maternal effects (such as mitochondrial genome inheritance and oocyte cytoplasmic factors [58,59,60]. Thus, the expression of hybrid vigor represents the combined effects of natural variation in regulatory sequences between parents and epigenetic mechanisms, providing a valuable model for studying the regulatory basis of adaptive evolution in fish.

In summary, the hybrid vigor observed in this hybrid system does not stem from a single factor, but rather represents a multi-level synergistic adaptive phenotype coordinated by non-additive gene expression. This molecular network enhances energy and nutrient acquisition efficiency through improved digestive system structure and function; redirects metabolism toward anabolic growth through reconfigured lipid metabolism and endocrine axes (GH/IGF and thyroid hormones); and potentially enhances physiological robustness and environmental adaptability via optimized stress response pathways (e.g., detoxification and hypoxia responses). This integrated physiological state, driven by genome-wide transcriptional reprogramming, systematically explains the synergistic advantages exhibited by the hybrid in growth rate, feed utilization, and survival rate.

It should also be noted that this experimental design has certain limitations. Each group was reared in a separate net cage. Although all cages were placed in the same pond to minimize environmental variation, no group had true replicate cages. This means that our statistical model could not fully distinguish between potential cage-specific effects, such as micro-environmental variations, and genetic effects. Therefore, while the intergroup differences observed in this study are primarily attributable to genetic factors, potential environmental influences may also be present.

## 5. Conclusions

This study demonstrates that hybridization between *M. armatus* and *M. favus* produced offspring with significant heterosis in terms of growth rate, survival and digestive capacity. This superiority originates from extensive non-additive gene expression in the hepatic transcriptome. These genes were significantly enriched in core pathways, including those involved in growth hormone synthesis, insulin secretion, and lipid metabolism. The coordinated upregulation of growth-promoting genes (e.g., *igf2* and *dio2*) and the downregulation of repressors (e.g., *socs1* and *igfbp2*) have established an efficient molecular network that regulates growth. Furthermore, the activation of stress-responsive pathways such as cytochrome P450 may underpin the observed enhancement in survival rate under culture conditions. By elucidating the mechanisms underlying heterosis in hybrid mastacembelid eels in multiple dimensions, this research provides a crucial theoretical foundation and practical strategies for elite breeding programs.

Despite these promising findings, this study has certain limitations that should be addressed in future research. Firstly, the specific roles of the identified non-additively expressed genes (e.g., *gskcb*, *elovl7*, *igf2*) in the heterosis network remain to be confirmed through targeted genetic experiments, such as gene knockout or overexpression. Secondly, the observed physiological advantages, particularly the enhanced environmental adaptability linked to stress-response pathways, require further investigation under real-world aquaculture conditions in order to assess their practical significance. Finally, while the reciprocal cross design (LT vs. TL) revealed maternal effects, the underlying molecular mechanisms, including the potential roles of mitochondrial inheritance or genomic imprinting, require further exploration. Future studies should also evaluate the long-term stability of heterosis and the reproductive capability of hybrid offspring across multiple generations, in order to assess their full breeding potential.

## Figures and Tables

**Table 1:** Intestinal parameters of different groups in zig-zag eel.

Group	LT	TL	TJ	LC
Fold Height/µm	427.72 ± 72.76 ^a^	318.31 ± 64.59 ^b^	244.86 ± 51.68 ^c^	267.53 ± 49.36 ^c^
Fold Width/µm	123.38 ± 42.55	114.75 ± 28.06	123.69 ± 31.60	106.77 ± 34.49
Fold density number/mm	5.86 ± 0.21 ^b^	6.36 ± 0.16 ^a^	4.13 ± 0.10 ^c^	5.35 ± 0.27 ^b^
Crypt Depth/µm	43.48 ± 13.56 ^a^	43.15 ± 8.09 ^a^	34.56 ± 3.60 ^b^	31.49 ± 5.24 ^b^
Fold Length/Crypt Depth	12.52 ±1.95 ^a^	9.37 ± 1.69 ^b^	7.11 ± 0.56 ^c^	8.62 ± 0.54 ^b^
Muscle Thickness/µm	108.96 ± 28.7 ^a^	66.29 ± 11.30 ^b^	41.05 ± 7.71 ^c^	52.38 ± 4.48 ^b^

*Note*: Values are presented as mean ± SD. Within each row, means with different superscript letters (a, b, c) are significantly different (*p* < 0.05, one-way ANOVA followed by Tukey’s HSD test).

**Table 2:** Summary statistics of transcriptome sequencing data.

Sample	Raw Reads (M)	Clean Reads (M)	Q20 (%)	Q30 (%)	GC (%)	Uniquely Mapping (%)	Total Mapping (%)
LC-2	86.55	85.89	98.48	95.67	49.7	64.14	69.12
LC-3	83.10	82.31	98.40	95.51	49.54	63.8	68.82
LC-5	118.13	116.98	98.34	95.37	49.58	62.92	68.55
LC-6	127.17	126.14	98.41	95.49	49.49	63.42	68.15
LT-1	74.76	74.31	98.84	96.67	48.93	62.18	66.71
LT-2	61.23	60.86	98.73	96.34	49.69	63.35	67.59
LT-3	133.13	131.88	98.35	95.39	49.52	62.55	67.12
LT-4	117.91	116.93	98.41	95.48	49.4	62.56	67.04
TJ-1	80.55	80.12	98.96	98.96	49.76	64.02	70.12
TJ-2	83.77	83.19	98.70	96.27	49.75	62.01	67.46
TJ-3	117.57	116.52	98.42	95.54	49.56	62.23	67.74
TJ-4	114.22	113.42	98.48	95.69	49.69	63.68	69.27
TL-5	88.91	88.38	98.81	96.58	49.19	62.38	67.1
TL-6	83.99	83.31	98.44	96.57	49.49	61.92	66.27
TL-8	77.42	76.83	98.44	95.59	49.65	61.69	67.12

## Data Availability

The data that support the findings of this study are openly available in the NCBI BioProject repository under accession number PRJNA1348105 at https://www.ncbi.nlm.nih.gov/bioproject/PRJNA1348105 (accessed on 23 October 2025).

## Supplementary Materials

The following supporting information can be downloaded at: https://www.mdpi.com/article/10.3390/ani16010011/s1, Figure S1: Phenotypes of four offspring groups at 90 days of age; Table S1: Feeding strategy for the offspring; Table S2: Nutritional composition of the eel feed; Table S3: Tukey HSD post-hoc test results for offspring growth data among four groups (F-value, degrees of freedom, and *p*-value); Table S4: Shapiro-Wilk test for normality of the growth data; Table S5: Results of the Tukey HSD post-hoc test for offspring digestive enzyme activity data among four groups (F-value, degrees of freedom, and *p*-value); Table S6: Shapiro-Wilk test for normality of the digestive enzyme activity data.
