# Supplementary material for: Growth Performance, Digestive Capacity, and Transcriptomic Analysis of the Hybrid Offspring of Mastacembelus armatus × Mastacembelus favus
Source: Animals (Basel). 2025 Dec 19;16(1):11. doi: 10.3390/ani16010011 (PMC12784677; doi:10.3390/ani16010011)
Supplement: Supplementary file 1 [file animals-16-00011-s001.zip › animals-4023292-supplementary.pdf]

Supplementary Materials:

Supplementary Table S1. Feeding strategy for the offspring

	Days Post-Hatching		
	7-15 Days	15-30 Days	30-90 Days
Feeding Protocol	Apparent satiation feeding		
Feed	*Artemia salina*	*Tubifex tubifex*	Eel formulated feed
Feed Source	Tianjin Yingwei Aquaculture Co., Ltd.	Hunan Red Worm Cultivation Base	Fujian Tianma Feed Co., Ltd.
Feeding Frequency	Twice daily		
Water Exchange & Waste Removal	Water was exchanged every three days; residual feed was removed 1 hour post-feeding.		

Supplementary Table S2. Nutritional composition of the eel feed

Ingredient (g/kg)	—
Gluten flour	38
Brown fish meal (Chile ORIZON S.A.)	170
White fish meal (American Seafoods Inc.)	287
White fish meal (Russia)	87
Fermented soybean meal	120
Extruded soybean	25
α-Starch	248.5
Choline chloride	2
Calcium biphosphate	10
Vitamin C	1
Vitamin complex	2
Mineral matter	5
Taurine	1
Lysine	2
Methionine	1.5
Ingredient (g/kg)	—
Total	1000
Nutrient levels (%)	—
Crude protein	43
Crude lipid	4
Crude ash	18
Crude fiber	4
Total phosphorus	1

Note: The basic diet composition and nutritional levels are provided by Fujian Tianma Feed Co., Ltd.

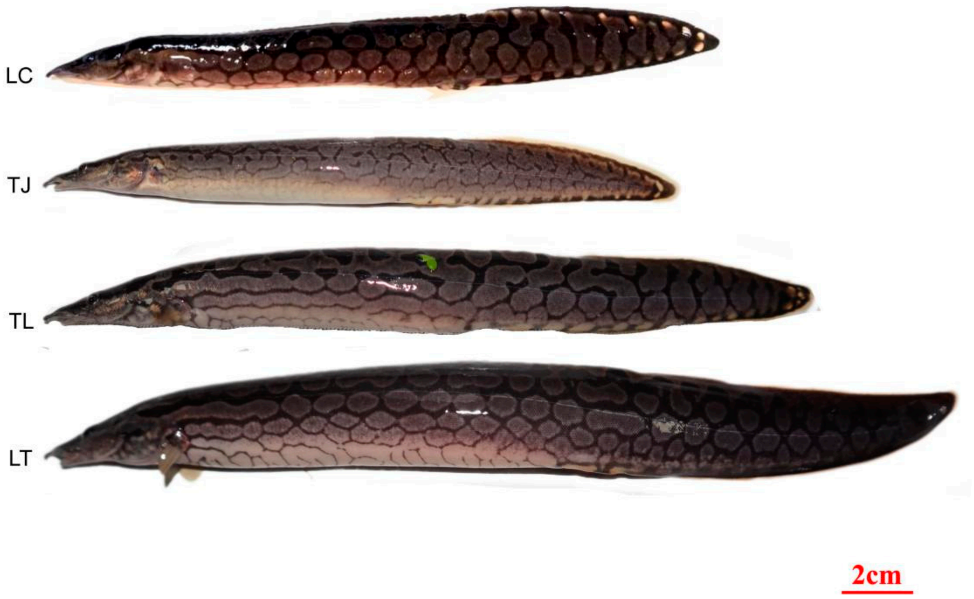

Supplementary Figure S1. Phenotypes of four offspring groups at 90 days of age

Supplementary Table S3. Tukey HSD post-hoc test results for offspring growth data among four groups (F-value, degrees of freedom, and p-value)

	p						dfB	dfW	F
	TL vs. LT	TL vs. LC	TL vs. TJ	LT vs. LC	LT vs. TJ	LC vs. TJ			
Length of 30-day-old fish	0.5533	>0.9999	0.1642	0.5667	0.5667	0.2427			
Length of 60-day-old fish	0.1502	0.2103	0.0001	0.4371	0.0025	<0.0001	3	56	51.98
Length of 90-day-old fish	0.003	0.0005	<0.0001	<0.0001	<0.0001	0.0028			
Weight of 30-day-old fish	0.6205	<0.0001	0.0001	<0.0001	0.0001	0.9927			
Weight of 60-day-old fish	0.6418	0.5166	0.0001	0.3573	0.0063	0.001	3	56	46.84
Weight of 90-day-old fish	<0.0001	0.0158	<0.0001	<0.0001	<0.0001	0.0083			
SGR_L	0.0001	<0.0001	0.0035	<0.0001	0.7577	<0.0001	3	56	42.05
SGR_W	0.0003	>0.9999	0.0015	0.0003	<0.0001	0.0015	3	56	22.74
survival rates	0.7871	0.0220	0.0105	0.0064	0.0033	0.9428	3	8	14.14

Note: The reported p-values are from post-hoc tests. The associated ANOVA summary includes: dfB (between-groups df), dfW (within-groups df), and the F-statistic (where F = MSB / MSW).

Supplementary Table S4. Shapiro-Wilk test for normality of the growth data

	W				p				Normality test
	TJ	LC	TL	LT	TJ	LC	TL	LT	
Length	0.9902	0.9988	0.9977	0.9839	0.8105	0.9343	0.9086	0.7571	Pass
Weight	0.8944	0.9711	0.8625	0.8550	0.3678	0.6737	0.2744	0.2539	Pass
SGRL	0.9349	0.9397	0.9426	0.9606	0.3230	0.3788	0.4167	0.7027	Pass
SGRW	0.9561	0.9255	0.9420	0.9255	0.6243	0.2333	0.4088	0.2333	Pass
survival rates	0.9992	0.9323	0.8489	0.9788	0.9465	0.4974	0.2375	0.7209	Pass

Note: W = test statistic (a quantitative score for normality fit); p = significance probability (probability of the data assuming normality).

Supplementary Table S5. Results of the Tukey HSD post-hoc test for offspring digestive enzyme activity data among four groups (F-value, degrees of freedom, and p-value)

	P						dfB	dfW	F
	TL vs. LT	TL vs. LC	TL vs. TJ	LT vs. LC	LT vs. TJ	LC vs. TJ			
Intestinal amylase	0.9203	0.0002	0.0004	0.0003	0.0008	0.7271	3	8	37.27
Liver amylase	0.3445	0.0025	0.0096	0.0004	0.0012	0.7002	3	8	24.38
Intestinal lipase	0.0368	<0.0001	<0.0001	<0.0001	<0.0001	0.9887	3	8	230.6
Liver lipase	0.0493	0.0257	0.0437	0.0006	0.0008	0.9796	3	8	20.8
gastric lipase	0.0047	0.2319	0.0618	0.0005	0.0002	0.7787	3	8	25.67
Intestinal trypsin	<0.0001	0.0005	<0.0001	<0.0001	<0.0001	0.2096	3	8	485.9
Liver trypsin	0.1684	0.0261	0.0035	0.0007	0.003	0.57	3	8	17.23
Pepsin	0.0002	0.0002	0.0015	<0.0001	<0.0001	>0.9999	3	8	48.1

Note: Enzyme activity was originally measured with four biological replicates per group. The outlying values were then trimmed uniformly across all groups.

Supplementary Table S6. Shapiro-Wilk test for normality of the digestive enzyme activity data

	W				p				Normality test
	TJ	LC	TL	LT	TJ	LC	TL	LT	
Intestinal amylase	0.9941	1.000	0.8493	0.9615	0.8537	0.9940	0.2385	0.6230	Pass
Liver amylase	0.9474	0.9217	0.8806	0.9968	0.5580	0.4585	0.3263	0.8921	Pass
Intestinal lipase	0.9994	0.9554	0.9566	0.8116	0.9530	0.5936	0.5990	0.1424	Pass
Liver lipase	0.9638	0.9661	1.000	0.9961	0.6343	0.6463	>0.9999	0.8802	Pass
gastric lipase	0.9998	0.9450	0.8337	0.9633	0.9757	0.5480	0.1978	0.6319	Pass
Intestinal trypsin	1.000	0.8776	1.000	0.8701	>0.9999	0.3173	>0.9999	0.2958	Pass
Liver trypsin	0.8086	0.7790	0.9421	0.9458	0.1353	0.0653	0.5357	0.5512	Pass
Pepsin	0.9965	1.000	0.9436	0.9436	0.8868	>0.9999	0.5421	0.5421	Pass

Note: W = test statistic (a quantitative score for normality fit); p = significance probability (probability of the data assuming normality).
